# Integrative transcriptomics and phenotyping uncover genetic networks controlling fruit quality in two strawberries (*Fragaria × ananassa*)

**DOI:** 10.3389/fpls.2025.1700348

**Published:** 2026-01-19

**Authors:** Yubo Chen, Hainan Liu, Ting Jiang, Kangjian Song, Xueming Zhang, Maojun Zhang, Huanyu Yao, Xiande Duan, Mingzheng Duan, Muhammad Junaid Rao

**Affiliations:** 1 Institute of Pomology, Jilin Academy of Agricultural Sciences, Gongzhuling, China; 2 Advanced Institute of Ecological Agriculture and Biodiversity on the Yunnan-Guizhou Plateau, Zhaotong University, Zhaotong, China; 3 State Key Laboratory for Development and Utilization of Forest Food Resources, Zhejiang A & F University, Hangzhou, Zhejiang, China

**Keywords:** breeding applications, fruit hardness, NAC transcription factors, polygalacturonases, soluble solids content, strawberry fruit quality, transcriptomics, WGCNA

## Abstract

Strawberry fruit quality is a complex trait governed by molecular mechanisms that undergo dynamic changes during ripening. This study investigated two varieties with contrasting phenotypes: ‘Monterey’, a firm-fleshed commercial cultivar, and ‘Three Princess’, a softer, sweeter variety. We analyzed five key developmental stages (green, white, transfer color, red, and overripe) to unravel the transcriptional basis of their divergent quality traits. RNA-seq revealed extensive differential gene expression (11,382 DEGs at the red stage, FDR < 0.05), with ‘Three Princess’ showing earlier activation of starch/sucrose metabolism and specific polygalacturonases (*Fxa1Ag102477*), correlating with its rapid softening and higher soluble solids content (SSC). WGCNA identified co-expression modules strongly linked to hardness, including *MElightsteelblue1* (positively correlated) and *MEantiquewhite1* (negatively correlated). Crucially, we found that firmness retention in ‘Monterey’ was associated not with the suppression of polygalacturonases but with the sustained high expression of key NAC transcription factors like *Fxa2Dg203497*, suggesting a potential transcriptional mechanism associated with delayed softening. Enriched pathways like galactose metabolism and fatty acid degradation further highlighted metabolic shifts driving texture and sweetness. Our findings demonstrate that varietal differences in fruit quality are determined by a stage-specific transcriptional interplay between cell wall-disassembling enzymes and their transcriptional regulators. These results provide candidate genes and regulatory networks for breeding strawberries with optimized texture and flavor, advancing our understanding of the complex molecular mechanisms governing strawberry fruit quality during ripening.

## 1 Introduction

Strawberry (*Fragaria × ananassa*) is a globally important fruit crop, prized for its vibrant color, sweet flavor, and delicate texture (Giampieri et al., 2014; Simpson, 2018; Fan and Whitaker, 2024). Commercial and consumer appeal largely depends on two key quality traits: fruit firmness and sweetness, the latter often assessed as soluble solid content (SSC) (Liu et al., 2023). Firmness influences postharvest shelf life, transport resilience, and sensory quality, while SSC, primarily composed of sugars, defines the fruit’s sweetness and overall palatability (Leisso et al., 2021). These traits are highly dynamic during fruit development and ripening, governed by a complex interplay of genetic, metabolic, and environmental factors. Understanding their molecular regulation is therefore crucial for breeding programs aimed at enhancing fruit quality and marketability.

Strawberry fruit development progresses through distinct physiological stages, each marked by specific biochemical and transcriptional changes. The transition from immature green fruit to ripe red fruit involves coordinated shifts in pigmentation, texture, and sugar accumulation (Leisso et al., 2021; Sánchez-Gómez et al., 2022). Early stages are characterized by cell division and expansion, accompanied by the accumulation of starch and organic acids. As the fruit enters the ripening phase, starch is hydrolyzed into soluble sugars, cell walls are remodeled, leading to softening, and anthocyanins are synthesized to impart the characteristic red color (Liu et al., 2023; López-Casado et al., 2023). The final overripe stage is marked by further softening and incipient senescence, which can lead to spoilage and economic losses (Quarshi et al., 2023; Priyadarshi et al., 2024). While these developmental transitions are well-documented phenotypically, the transcriptional networks driving them, particularly those governing texture and sweetness, remain incompletely characterized.

Recent advances in transcriptomics have shed light on the genetic regulation of strawberry ripening (Sánchez-Gómez et al., 2022). RNA sequencing (RNA-seq) studies have identified key differentially expressed genes (DEGs) involved in cell wall degradation, hormone signaling, and sugar metabolism (Li et al., 2024; Wang et al., 2025). For instance, polygalacturonases and expansins, which degrade pectin and loosen cell walls, are upregulated during softening, while sucrose synthase and invertases modulate sugar accumulation (Quarshi et al., 2023; Yang et al., 2024; Wang et al., 2025). Transcription factors (TFs) such as those in the MADS-box, NAC, and MYB families have emerged as central regulators of these processes (Liu et al., 2022; Sánchez-Gómez et al., 2022; Zhang et al., 2024). Notably, FvMYB79 (involved in transcriptional activation of FvPME38) and *FaRIF* (a NAC gene) have been linked to strawberry fruit softening. Similarly, PavNAC56 regulates ripening in sweet cherry fruit (Qi et al., 2022). Despite these insights, most studies have focused on single varieties or limited developmental stages, leaving gaps in our understanding of how transcriptional dynamics vary across genotypes and contribute to divergent fruit quality traits.

A critical knowledge gap lies in the comparative transcriptomics of strawberry varieties with contrasting texture and sweetness profiles. While firmness and SSC are known to vary significantly among cultivars, the genetic basis for these differences remains poorly explored. For example, some varieties retain firmness during ripening (a desirable trait for transport and storage), while others soften rapidly but achieve higher sugar content (Liu et al., 2023; Quarshi et al., 2023). Additionally, most transcriptomic studies have concentrated on the red ripe stage, overlooking earlier transitions (e.g., green to white stages) that may prime the fruit for subsequent ripening events. The role of co-expression networks in modulating fruit quality has been under-investigated, despite their potential to fine-tune gene expression during development (Moyano et al., 2018; Liu et al., 2023; Martín-Pizarro et al., 2025). Addressing these gaps requires a comprehensive, stage-resolved comparison of varieties with distinct phenotypic traits.

This study bridges these gaps by integrating phenotypic and transcriptomic analyses of two strawberry varieties such as ‘Three Princess’ and ‘Monterey’, which exhibit contrasting hardness and SSC patterns. ‘Monterey’ is a firm-fleshed commercial variety, while ‘Three Princess’ is a softer, sweeter cultivar, providing an ideal system to dissect the genetic determinants of fruit quality. Using RNA-seq, we identified DEGs, enriched pathways, and co-expression networks associated with firmness and SSC across five developmental stages (green, white, transfer color, red, and overripe). We focused on key TF families and enzymatic genes to elucidate their stage-specific roles in ripening regulation. Our objectives were threefold: (1) to characterize the phenotypic progression of hardness and SSC in both varieties, linking these traits to distinct developmental phases; (2) to unravel the global transcriptomic shifts driving these phenotypic changes, with emphasis on cell wall modification and sugar metabolism pathways; and (3) to identify candidate genes and regulatory networks that differentiate ‘Monterey’ and ‘Three Princess’, offering targets for marker-assisted breeding. By combining high-resolution phenotyping with multi-stage transcriptomics, this work provides insights into the genetic control of strawberry fruit development. The findings not only advance fundamental knowledge of ripening but also have practical implications for improving strawberry quality and postharvest performance.

## 2 Materials and methods

### 2.1 Plant materials and growth conditions

The study was conducted in 2022 under controlled conditions in a plastic greenhouse at the Fruit Tree Research Institute of Jilin Academy of Agricultural Sciences (Gongzhuling City, China). Two everbearing strawberry (*Fragaria × ananassa*) varieties were selected for analysis: “Three Princess (Gongzhu),” a self-bred cultivar developed by Jilin Academy of Agricultural Sciences, and “Monterey,” a commercially important variety originating from the United States. Plants were cultivated using an H-type frame system with a standardized substrate mixture composed of black soil, peat, perlite, and cow dung in a 4:4:1:1 ratio (v/v). Throughout the growth period, plants were maintained under optimal conditions using an integrated drip irrigation and fertilization system to ensure consistent nutrient and water supply.

### 2.2 Sample collection and phenotypic measurements

Fruit samples were harvested at five developmental stages: 9 days after flowering (DAF; October 11, 2022, green stage), 18 DAF (October 20, white stage), 25 DAF (October 27, transfer color stage), 32 DAF (November 4, red stage), and 39 DAF (November 11, overripe stage). These stages were selected to capture key transitions in fruit maturation, from early growth to full ripening and senescence. Three biological replicates per stage and variety were analyzed. Each biological replicate consisted of a pool of 10 randomly selected fruits. All phenotypic measurements were performed on the day of harvest at room temperature (approximately 23°C). Fruit firmness was quantified using a GY-4 digital fruit firmness tester (Zhejiang Top Cloud-Agri Technology Co., Ltd., China), with measurements recorded in Newtons (N) to evaluate textural changes across developmental stages. Fruits were categorized as: very firm (>10 N), firm (5–10 N), soft (1–5 N), and very soft (<1 N), corresponding to the green, white, transfer color, and overripe stages, respectively. Soluble solid content (SSC) was measured using a handheld refractometer (Atago PAL-1, Japan). For SSC analysis, fresh fruit juice was extracted from homogenized samples, and measurements were expressed as °Brix.

### 2.3 RNA-seq analysis

Total RNA was extracted from strawberry fruit samples using the RNAprep Pure Plant Kit (Tiangen, Beijing, China) and quantified for purity (NanoDrop 2000; Thermo Fisher Scientific) and integrity (RNA Nano 6000 Assay Kit, Agilent 2100 Bioanalyzer) (1). Sequencing libraries were prepared from 1 μg total RNA using the NEBNext Ultra™ RNA Library Prep Kit (NEB #E7530) with poly-A selection and fragmentation, followed by cDNA synthesis, adapter ligation, and PCR amplification. Paired-end sequencing (150 bp) was performed on an Illumina NovaSeq 6000 platform.

Clean reads were generated by removing adapters, low-quality bases (Q < 20), and poly-N sequences using Trimmomatic v0.39. High-quality reads were aligned to the *Fragaria × ananassa* reference genome (*Fragaria_x_ananassa*.farr1.genome.fa; GDR database) using HISAT2 v2.2.1 with default parameters (Kim et al., 2015). The raw sequencing data generated in this study have been deposited in the China National GeneBank DataBase (CNGBdb) under accession number CNP0007866 and are publicly accessible at https://db.cngb.org/data_resources/project/CNP0007866/. Transcript assembly was performed with StringTie v2.1.4. A raw read count matrix for each gene was generated from the StringTie output, which was used as input for differential expression analysis. Gene expression levels were also calculated as FPKM (Fragments Per Kilobase Million) for visualization and other analyses. Differential expression analysis was performed using DESeq2 v1.30.1 on the raw count data (FDR < 0.05, |log2FC| ≥ 1). All samples had biological replicates, and DESeq2 was used consistently throughout the study. All DEGs are represented in the **Supplementary Material**. Functional enrichment of DEGs was analyzed via GOseq v1.42.0 (Gene Ontology) and KOBAS v3.0 (KEGG pathways), with significance thresholds of FDR < 0.05.

### 2.4 Weighted gene co-expression network analysis

Based on gene expression data obtained from RNA-seq, WGCNA was performed to construct gene co-expression networks, aiming to identify gene regulatory modules associated with fruit quality traits. The specific procedures are as follows:

#### 2.4.1 Gene filtering and data preprocessing

The raw FPKM matrix was subjected to quality filtering: genes with FPKM ≥ 1 in at least 3 samples were retained to ensure inclusion of reliably expressed genes. To focus on genes with biologically meaningful expression dynamics across development, we further applied a coefficient of variation (CV) cutoff of ≥ 0.5. This threshold was selected to enrich for genes showing moderate-to-high variability in expression across samples, which is more likely to reflect stage-specific or genotype-dependent regulation rather than stochastic noise. The filtered gene expression matrix was standardized using Z-score transformation to eliminate systematic biases between samples.

#### 2.4.2 Co-expression network construction

Network analysis was conducted using the WGCNA package (v1.72-1) in R. An unsigned weighted co-expression network was constructed. The optimal soft-thresholding power (β) was set to 17 based on an analysis of scale-free topology fit, which achieved a scale-free model fit index (R²) > 0.85 (**Supplementary Figure S1**). Based on this threshold, the weighted adjacency matrix between genes was calculated and converted into a topological overlap matrix (TOM) to quantify the strength of co-expression associations between genes.

#### 2.4.3 Module identification and naming

Genes were clustered using the Dynamic Hybrid Tree Cut algorithm based on the dissimilarity coefficient (1-TOM) of the TOM matrix. The minimum module size was set to 30 to ensure each module contained a sufficient number of genes for biological significance. The minimum height for merging modules was set to 0.25 to merge modules with highly similar expression patterns. The resulting co-expression modules were named with random colors (e.g., MElightsteelblue1, MEantiquewhite1, etc.), and the module eigengene (ME) of each module was calculated as a representative of the module’s expression profile.

#### 2.4.4 Correlation analysis between modules and phenotypes

Pearson correlation coefficients between each module eigengene and fruit phenotypic data (firmness, soluble solids content, etc.) were calculated to screen modules significantly associated with target traits (P < 0.05). For each associated module, hub genes within the module were identified by calculating the correlation between genes and module eigengenes (module membership, MM) and between genes and phenotypes (gene significance, GS). Genes with MM > 0.8 and GS > 0.6 were selected as hub genes, with a focus on key modules related to fruit firmness (e.g., MElightsteelblue1 and MEantiquewhite1).

### 2.5 Statistical analysis

Multivariate analytical approaches were employed to examine the expression patterns of firmness characteristics throughout various developmental phases. Prior to analysis, transcriptomic datasets underwent Z-score standardization; statistical significance was assessed through Student’s t-test with false discovery rate adjustment (P < 0.05) (Colquhoun, 2014), while biological significance was determined using fold-change thresholds (|log2FC| ≥ 1, corresponding to FC ≥ 2 or ≤ 0.5) (Fraga et al., 2010). Hierarchical cluster analysis (HCA) and principal component analysis (PCA) were conducted to discern inter-group variations while reducing intra-group variability, utilizing OmicShare analytical tools, an open-access online data analysis platform (http://www.omicshare.com/tools) (Mu et al., 2024). Visual presentations and standard error computations were prepared using Microsoft Excel (Redmond, WA, USA). Statistix 8.1 software (Tallahassee, FL, USA) facilitated statistical evaluations of biochemical variables, with all assays performed in triplicate. Variations in firmness and sugar concentrations between strawberry samples were assessed through the Least Significant Difference (LSD) analysis (p < 0.05).

Venn diagram construction was accomplished using the EVenn web-based platform (https://www.bic.ac.cn/EVenn/#/). Pearson correlation analyses were executed via the OmicShare computational platform (http://www.omicshare.com/tools) (Mu et al., 2024).

## 3 Results

### 3.1 Phenotypic changes during fruit development

**Figure 1:**
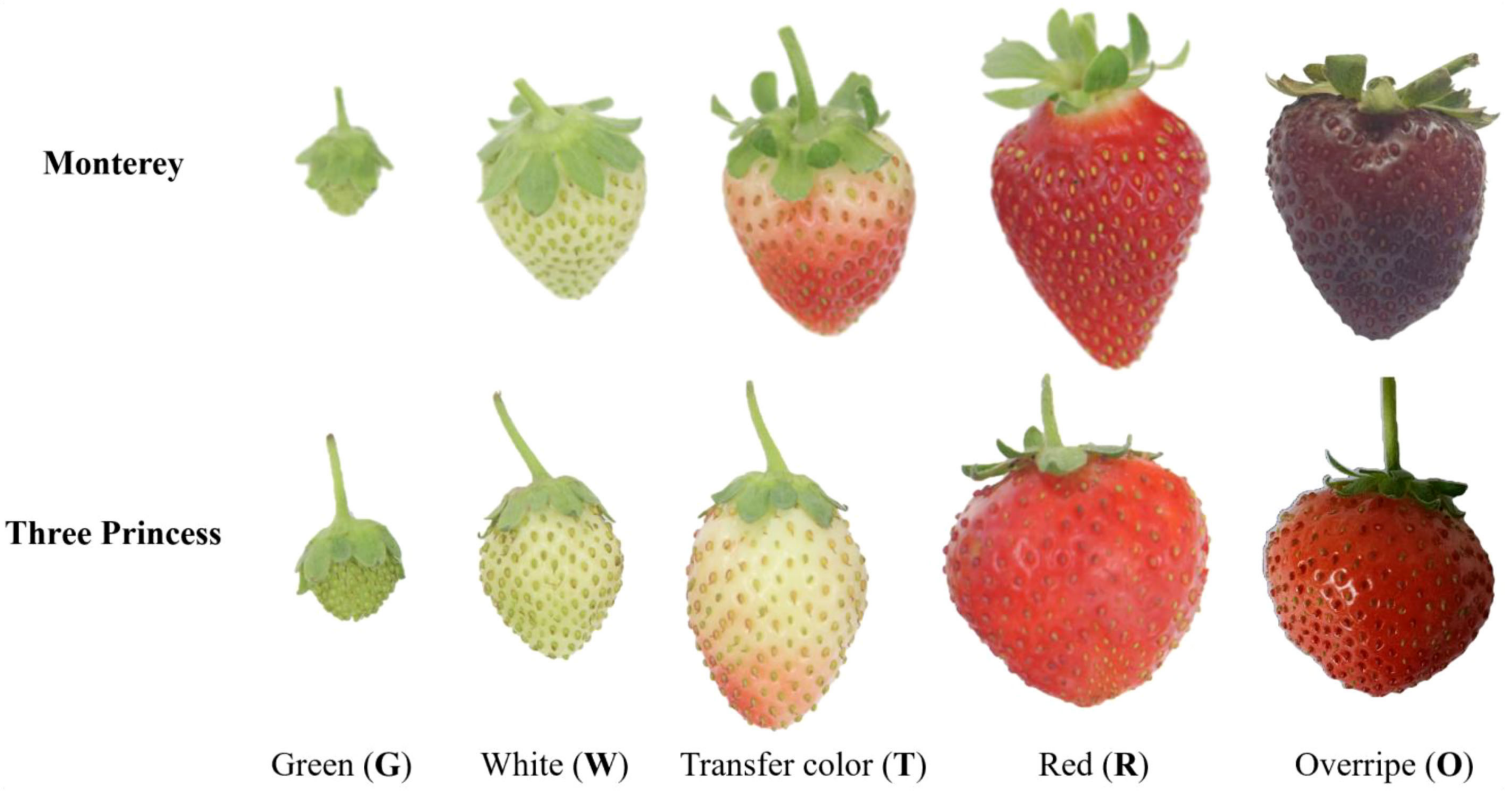
Phenotypic progression of two strawberry varieties across developmental stages. Representative images of Monterey and Three Princess fruits at four stages: green (G), white (W), transfer color (T), red (R), and overripe (O).

The phenotypic changes observed during the development of the two strawberry varieties, Monterey and Three Princess, were categorized into five distinct stages: green (G), white (W), transfer color (T), red fruit (R) and overripe (O) (**Figure 1**). Initially, the fruits appeared as small, hard, and uniformly green (G). As they matured, the size increased and color transitioned to a pale white (W stage), accompanied by a slight softening (**Figure 1**). The next stage (T) marked a critical shift, with the fruits developing a mix of pink and red hues, indicating the onset of ripening (**Figure 1**). Next, the berries reached maturity (R), exhibiting a vibrant red color, less hardness, and juicy texture (**Figure 1**). Finally, in the overripe stage, the fruits displayed further softening, darker pigmentation, and incipient signs of senescence, including slight shriveling and loss of turgor (**Figure 1**).

**Figure 2:**
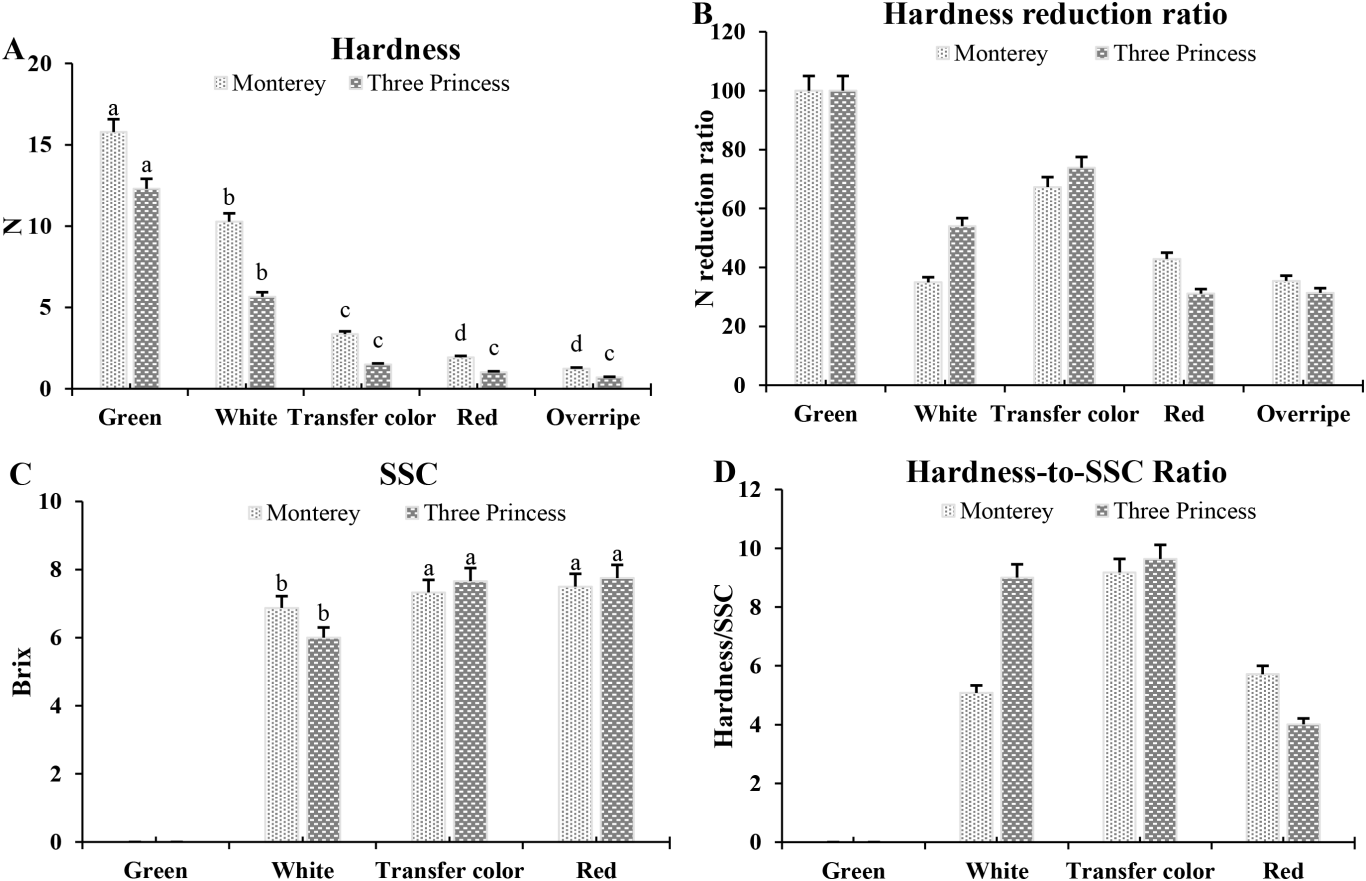
Hardness and their reduction ratios of ‘Monterey’ and ‘Three Princess’ strawberries across five developmental stages (Green, White, Transfer Color, Mature, Overripe). **(A)** Hardness values (N), **(B)** Hardness reduction ratios, **(C)** soluble sugar content (°Brix), and **(D)** Hardness-to-SSC ratio. Vertical bars represent SE± n=3. Small letters represent variations in firmness and sugar concentrations between strawberry samples were assessed through the Least Significant Difference (LSD) analysis (p < 0.05).

Hardness measurements of ‘Monterey’ and ‘Three Princess’ strawberries revealed stage-dependent textural changes (**Figure 2A**). Both varieties showed peak firmness in the green stage (‘Monterey’: 15.78 N; ‘Three Princess’: 12.29 N). Initial softening occurred during the white stage (‘Monterey’: 10.27 N; ‘Three Princess’: 5.65 N). The most significant texture change happened at the transfer color stage (‘Monterey’: 3.36 N; ‘Three Princess’: 1.48 N), paralleling anthocyanin (red color) accumulation and cell wall modification. Notably, ‘Three Princess’ exhibited accelerated softening during this transition despite its earlier firmness advantage. At full ripeness (red stage), both cultivars reached minimal firmness (‘Monterey’: 1.92 N; ‘Three Princess’: 1.02 N). Overripening further reduced firmness, with ‘Three Princess’ (0.7 N) becoming significantly softer than ‘Monterey’ (1.24 N). These quantitative changes demonstrate: (1) ripening-associated textural modification occurs in discrete phases, (2) ‘Three Princess’ undergoes more dramatic softening despite initial firmness, and (3) varietal differences persist through late developmental stages.

Hardness reduction ratios revealed distinct softening patterns between varieties (**Figure 2B**). The hardness reduction ratio was calculated for each developmental stage as the percentage of hardness retained compared to the green stage: (hardness at a later stage/hardness at the green stage) × 100% (This metric normalized the softening progression against the initial firmness of each variety). Both started at 100% firmness (green stage) but diverged at the white stage (‘Three Princess’: 54.03%; ‘Monterey’: 34.9%). During transfer color, ‘Three Princess’ showed greater proportional firmness retention (73.81% vs. 67.28%) despite faster phenotypic ripening (**Figure 1**). At maturity, ‘Monterey’ maintained higher firmness (42.86% vs. 31.08%), consistent with its commercial firmness. These trends demonstrate that: (1) softening rates vary by variety and stage, (2) phenotypic progression aligns with textural changes, and (3) ‘Monterey’ retains structural integrity longer, even when appearing equally ripe.

Soluble solid content (SSC, °Brix) increased progressively, at the white fruit stage, SSC measured 6.0 and 6.875°Brix for ‘Three Princess’ and ‘Monterey’, respectively (**Figure 2C**). By the transfer color stage, values rose to 7.66 and 7.33°Brix, with ‘Three Princess’ maintaining its lead. At mature stage, ‘Monterey’ reached 7.5°Brix and ‘Three Princess’ peaked at 7.75°Brix (**Figure 2C**). Although the absolute differences in SSC were modest, they were statistically significant and are consistent with the established sensory impact of small soluble solid variations in strawberry, where differences of 0.5–1.0°Brix can influence perceived sweetness and consumer preference (Lee et al., 2018; Luo et al., 2020; Fan et al., 2021). These quantitative results demonstrate that ‘Three Princess’ accumulates sugars earlier and achieves higher sweetness than ‘Monterey’. The hardness-to-SSC results showed that ‘Monterey’ had lower ratios in white (5.08) and transfer color (9.18) stages, indicating firmer texture relative to sweetness, while ‘Three Princess’ exhibited higher ratios (9 and 9.64 respectively) (**Figure 2D**). Notably, both varieties reached comparable ratios at maturity (‘Monterey’: 5.71; ‘Three Princess’: 4.01), suggesting ‘Three Princess’ maintains better textural stability despite its higher SSC (**Figure 2D**). This better preservation of textural stability in ‘Three Princess’ despite higher SSC may be linked to its distinct cell wall remodeling program, characterized by the early but coordinated expression of specific polygalacturonases (e.g., *Fxa1Ag102477*) rather than a broad, unregulated degradation, as well as the potential role of other cell wall strengthening genes identified in the WGCNA.

### 3.2 Global transcriptome changes

High-quality RNA-seq data were obtained for all 30 samples, with total 194.13 Gb clean reads were obtained ranging from 19.1–25.0 million per sample (mean: 21.6 million) and Q30 scores consistently >91.89% (mean: 94.2%), confirming robust sequencing accuracy (**Table 1**). GC content remained stable across developmental stages (46.3–47.0%), with minor varietal differences (Three Princess: 46.4–46.9%; Monterey: 46.3–47.0%).

**Table 1:** Statistics of transcriptomic sequencing data of 30 strawberry samples.

Serial No.	Samples	Clean reads	Clean bases	GC Content	%≥Q30
1	3G-1	25,985,372	7,780,595,516	46.88%	94.47%
2	3G-2	22,757,533	6,814,934,966	47.03%	94.55%
3	3G-3	22,167,532	6,636,588,596	46.87%	93.70%
4	MG1	19,661,983	5,885,004,966	46.64%	93.98%
5	MG2	22,739,925	6,808,610,464	46.61%	94.72%
6	MG3	21,110,617	6,320,900,438	46.58%	94.13%
7	MO1	20,301,973	6,079,114,418	46.63%	93.94%
8	MO2	21,256,223	6,363,831,222	46.53%	94.89%
9	MO3	20,476,257	6,131,352,824	46.44%	94.76%
10	MR1	21,423,532	6,413,228,510	46.65%	94.68%
11	MR2	20,022,900	5,995,527,540	46.37%	92.98%
12	MR3	23,390,891	7,003,028,298	47.00%	94.07%
13	MT1	19,131,936	5,729,224,012	46.50%	94.23%
14	MT2	23,534,200	7,047,492,736	46.34%	94.42%
15	MT3	22,434,829	6,717,813,858	46.37%	93.70%
16	MW1	20,974,375	6,277,100,318	46.57%	93.54%
17	MW2	20,813,818	6,231,678,596	46.90%	94.70%
18	MW3	21,693,880	6,496,123,268	46.81%	94.48%
19	3O-1	20,776,538	6,219,478,732	46.45%	94.91%
20	3O-2	24,793,881	7,423,529,338	46.34%	94.83%
21	3O-3	21,080,543	6,312,232,638	46.32%	94.96%
22	3R-1	23,525,756	7,043,696,142	46.56%	94.53%
23	3R-2	23,275,436	6,969,482,974	46.59%	91.89%
24	3R-3	21,394,235	6,405,640,448	46.51%	94.76%
25	3T-1	19,050,780	5,704,533,674	46.38%	94.42%
26	3T-2	20,873,722	6,250,243,698	46.45%	93.42%
27	3T-3	22,033,036	6,596,594,166	46.48%	94.82%
28	3W-1	21,044,704	6,301,101,690	46.81%	94.55%
29	3W-2	20,455,639	6,125,078,970	46.69%	94.84%
30	3W-3	20,210,694	6,050,714,190	46.68%	94.64%

Green ‘G’, White ‘W’, Transfer Color ‘T’, Red ‘R’, Overripe ‘O’) and Three Princess = ‘3’; Monterey = ‘M’.

**Figure 3:**
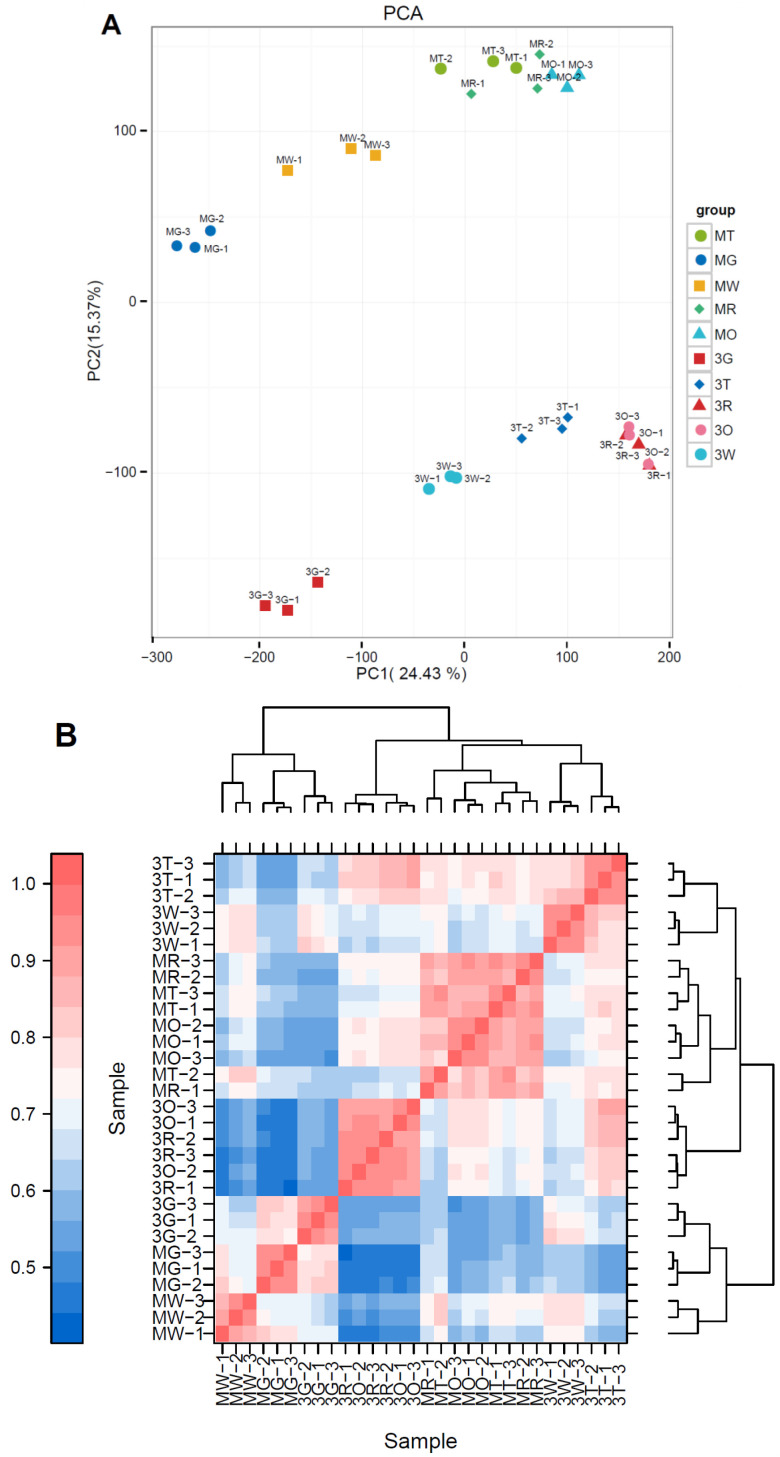
PCA plot and correlation analysis showing sample clustering by stage and variety. **(A)** PCA of strawberry transcriptomes across five developmental stages, **(B)** Spearman’s correlation heatmap of 30 strawberry samples. Color scale (0.5–1.0, blue to red) represents pairwise R values between transcriptomes. Clustering highlights stage-specific groups (Green ‘G’, White ‘W’, Transfer Color ‘T’, Red ‘R’, Overripe ‘O’) and varietal differences (Three Princess = ‘3’; Monterey = ‘M’).

Principal component analysis (PCA) of transcriptomic data revealed clear varietal and developmental segregation (**Figure 3A**). The first two principal components (PC1: 24.43%; PC2: 15.37%) effectively captured 39.8% of total variation, with Monterey samples clustering in the upper quadrant and Three Princess in the lower quadrant, demonstrating fundamental transcriptomic divergence between varieties. Developmental progression followed distinct trajectories: while both varieties showed orderly stage-wise separation along PC2, Three Princess exhibited greater dispersion during transfer color (T) to mature (R) transitions, suggesting more dynamic gene expression shifts. The Spearman’s correlation heatmap revealed distinct transcriptional patterns across developmental stages of two varieties (**Figure 3B**). Replicates within each stage-variety group showed high correlation coefficients (r > 0.85), confirming experimental reproducibility (**Figure 3B**).

### 3.3 Transcriptomic analysis of DEGs

Transcriptomic comparisons of differential expressed genes (DEGs) revealed dynamic gene expression shifts across strawberry development, with the mature fruit stage (R3 vs MR) showing the most extensive reprogramming (11,382 DEGs) (**Table 2**). Notably, up-regulated genes consistently outnumber down-regulated genes in all comparisons (e.g., 6,355 vs 5,027 in R3 vs MR), suggesting activation of ripening pathways dominates over repression (**Table 2**). The overripe phase (O3 vs MO) exhibited near-balanced up/down-regulation (4,743 vs 4,495), reflecting its transitional nature, while early developmental transitions (G3 vs MG) showed stronger up-regulation bias (4,922 up vs 3,969 down). In all different comparison groups ‘Three Princess’ showed maximum number of DEGs up regulated as compared to the ‘Monterey’ variety in their respective developmental stages (**Table 2**). These patterns align with the progressive activation of cell wall modification and secondary metabolism genes during ripening.

**Table 2:** Differential expressed genes identified in all strawberry sample groups.

Serail no.	DEG set	DEG number	Up regulated	Downregulated
1	3G vs MG	8,891	4,922	3,969
2	3O vs MO	9,238	4,743	4,495
3	3R vs MR	11,382	6,355	5,027
4	3T vs MT	8,285	4,267	4,018
5	3W vs MW	8,086	4,402	3,684

Green ‘G’, White ‘W’, Transfer Color ‘T’, Red ‘R’, Overripe ‘O’) and Three Princess = ‘3’; Monterey = ‘M’.

**Figure 4:**
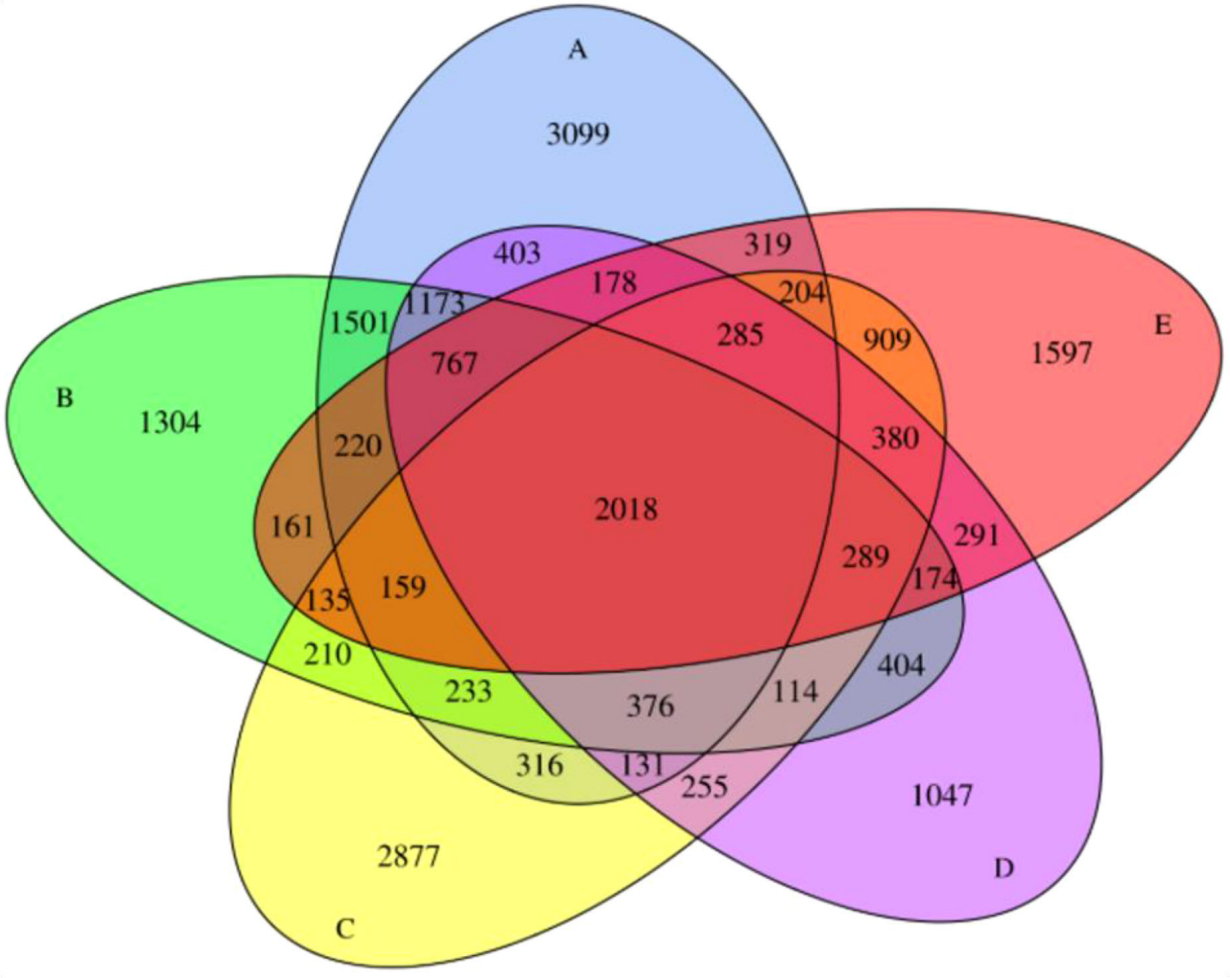
Venn diagram of differentially expressed genes among stage and variety. Circles represent comparisons: **(A)** (3R vs MR, blue), **(B)** (3O vs MO, green), **(C)** (3G vs MG, yellow), **(D)** (3T vs MT, purple), and **(E)** (3W vs MW, red). Intersection areas scale with gene counts, with key overlaps labeled. The central pentagon (gray) marks 2018 genes differentially expressed in all conditions. Data derived from RNA-seq of three biological replicates per stage (FDR<0.05, |log2FC|≥1).”.

Venn analysis of DEGs across five developmental comparisons revealed both stage-specific and shared regulatory networks (**Figure 4**). The red fruit transition (3R_vs_MR) contained the largest unique DEG set (3,099 genes), highlighting the extensive transcriptomic reprogramming occurring during final ripening (**Figure 4**). Notably, 2018 conserved DEGs appeared across all comparisons, representing a core set of ripening regulators—including known cell wall modifiers and sugar metabolism genes. The overripe phase (3O_vs_MO) shared the greatest overlap with other stages (1,501 common DEGs with 3R_vs_MR) while showing 1304 unique DEGs, suggesting it serves as a transcriptional pivot point (**Figure 4**). Surprisingly, early development green stage (3G_vs_MG) showed 2877 unique DEGs set, indicating distinct regulatory programs initiate developmental stage (**Figure 4**). Moreover, the transfer color (T) stage (3T vs MT) and white stage (3W vs MW) showed 1047 and 1597 unique set of DEGs respectively (**Figure 4**). Volcano plots visualizing the distribution of these DEGs are provided in **Supplementary Figure S2**. This transcriptional divergence highlights how discrete genetic programs are sequentially activated to drive specific physiological transitions during strawberry development.

### 3.4 Results of KEGG pathway enrichment analysis

**Figure 5:**
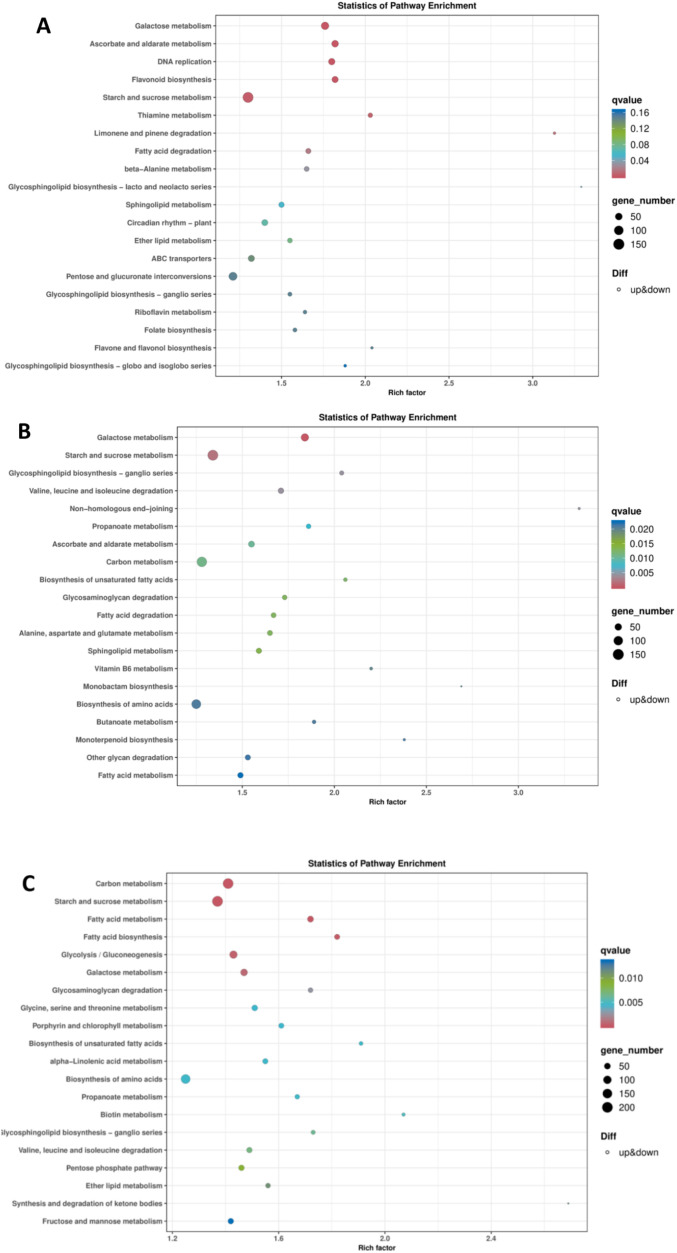
KEGG pathway enrichment on DEGs-Bubble chart. **(A)** KEGG enrichment of green stage among ‘Three Princess’ (3) and ‘Monterey’ (M) strawberries (3G vs MG), **(B)** KEGG enrichment of transfer color stage (3T vs MT), **(C)** KEGG enrichment of red stage (mature fruits) (3R vs MR) Dot size represents number of genes in pathway; color signifies significance (blue: most, yellow: least); position indicates enrichment strength on y-axis. Smaller q-values mean more reliable results.

The KEGG enrichment analysis of green (G) stage comparisons revealed significant activation of metabolic pathways during early development of ‘Three Princess’ (3) and ‘Monterey’ (M) strawberries (3G vs MG) (**Figure 5A**). The enriched pathways included starch and sucrose metabolism and flavonoid biosynthesis, indicating coordinated sugar accumulation and secondary metabolite production. Notably, pentose and glucuronate interconversions (critical for cell wall remodeling) showed moderate enrichment. The high gene counts (>100) in starch/sucrose and flavonoid pathways suggest these are dominant processes in green-to-mature transitions (**Figure 5A**). The KEGG analysis of transfer color (T) stages revealed distinct metabolic priorities between two strawberries (3T vs MT) (**Figure 5B**). Galactose metabolism and starch/sucrose metabolism emerged as the most enriched pathways, reflecting active sugar interconversions during pigmentation onset. Surprisingly, fatty acid degradation (q = 0.010) and branched-chain amino acid metabolism (valine/leucine degradation) showed significant activation, suggesting alternative energy mobilization alongside carbohydrate use (**Figure 5B**). Notably, Three Princess exhibited stronger enrichment in secondary metabolite pathways (monoterpenoid biosynthesis, glycosaminoglycan degradation) compared to Monterey, potentially explaining its more vivid pigmentation phenotype.

The KEGG analysis of red-stage (mature fruits) strawberries revealed a pronounced shift toward energy metabolism, with Three Princess (3R) exhibiting stronger activation of carbon metabolism (Rich Factor = 2.4) and starch/sucrose conversion than Monterey (MR) (**Figure 5C**). Key pathways like glycolysis/gluconeogenesis and fatty acid degradation were significantly enriched, reflecting the fruit’s transition to a high-energy state during final ripening. Notably, Three Princess showed unique upregulation of α-linolenic acid metabolism (linked to aroma volatile production) and porphyrin metabolism (associated with chlorophyll breakdown), aligning with its faster color transition phenotype (**Figures 1**, **5C**). The parallel enrichment of amino acid biosynthesis and pentose phosphate pathway suggests coordinated carbon and nitrogen flux to support flavor and pigment accumulation.

### 3.5 Genes screening related to strawberry hardness by weighted gene co-expression network analysis

**Figure 6:**
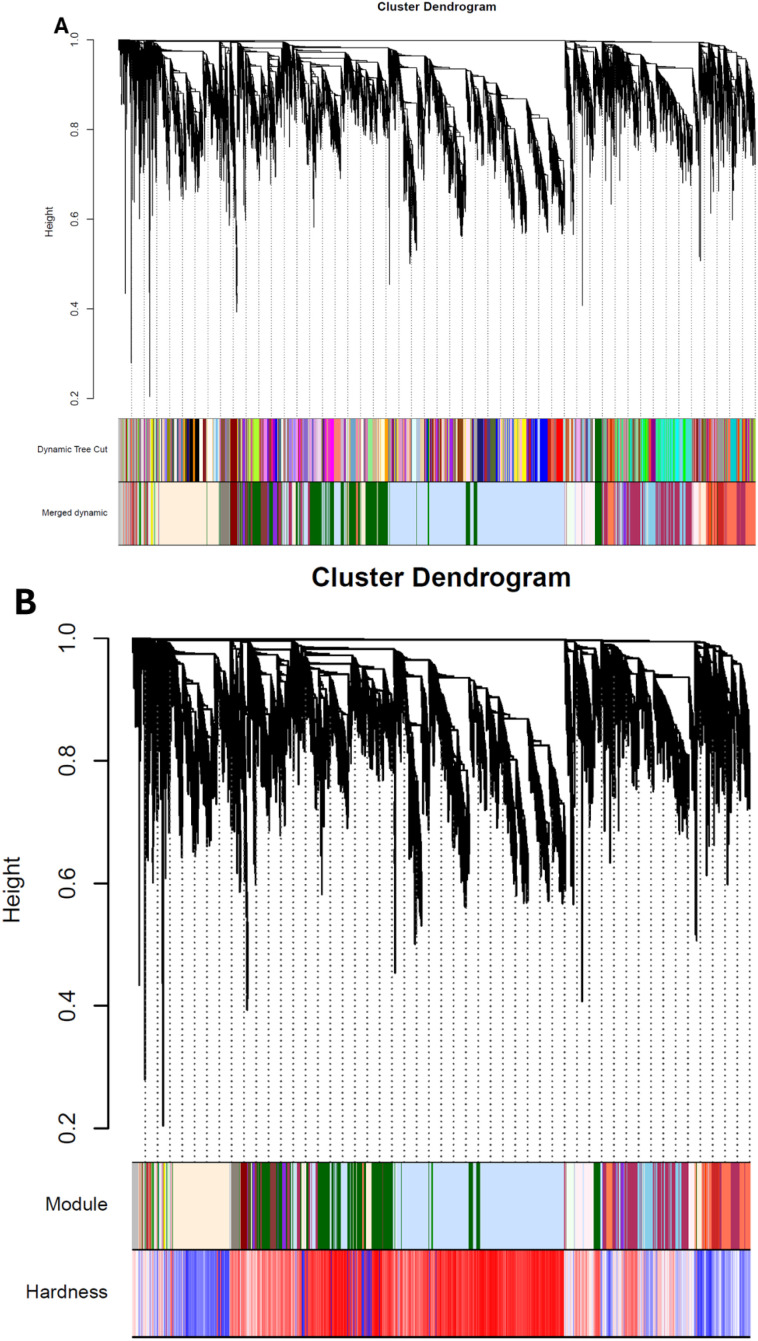
Hierarchical clustering dendrogram and network construction of (co-expressed) differentially expressed genes in different strawberry stages associated with hardness traits. **(A)** Cluster dendrogram and module assignment of co-expressed genes. The upper panel shows the hierarchical clustering of genes (y-axis: height representing the dissimilarity between two nodes, among genes). The middle panel displays the initial module assignments from dynamic tree cutting (colored bands), while the lower panel presents the final merged modules with similar expression patterns according to module similarity. Module colors are arbitrary and distinct for visualization purposes. **(B)** The clustering shows gene clustering based on topological overlap dissimilarity (y-axis: height). Module assignments are indicated by color bars beneath the dendrogram. The adjacent heatmap (lowerside) displays correlation values between each module and the Hardness trait (x-axis: modules, y-axis: trait), with color intensity representing correlation strength (blue: negative, red: positive).

**Figure 6A** presents a hierarchical clustering dendrogram of genes (greater than two-fold) based on their co-expression patterns, visualized through a tree structure where branch lengths represent the degree of dissimilarity (height) between gene clusters. Below the dendrogram, colored bands indicate the initial module assignments from dynamic tree cutting, where distinct colors correspond to different gene modules identified through the analysis. The lower section displays the final merged modules after optimization, showing how closely related modules were combined to reduce redundancy and refine the gene network groupings (**Figure 6A**). This visualization provides an overview of the gene clustering process, from initial partitioning to the consolidated module structure used for downstream analysis.

**Figure 6B** shows hierarchical clustering dendrogram of co-expressed genes alongside their association with the phenotypic trait *Hardness* in strawberry fruits. The dendrogram reveals distinct gene modules (indicated by color blocks) grouped based on topological overlap dissimilarity, with branch heights representing the degree of transcriptional divergence. Notably, one module (labeled in red) shows a strong negative correlation with hardness, suggesting its genes are significantly downregulated as fruit firmness decreases during ripening. Conversely, a smaller module (blue) exhibits a positive correlation, potentially representing cell wall reinforcement genes active in early developmental stages (**Figure 6B**). The clustering pattern aligns with the phenotypic data (**Figures 1**, **2**), where hardness declines sharply during the transfer color (T) to red (R) transition, implicating these modules in stage-specific textural regulation. Key genes within these modules include expansions, polygalacturonases, and NAC transcription factors, consistent with their known roles in cell wall disassembly and ripening. This integrative analysis highlights how coordinated gene expression dynamics underpin hardness variation, providing candidate targets for breeding firmer strawberry varieties.

**Figure 7:**
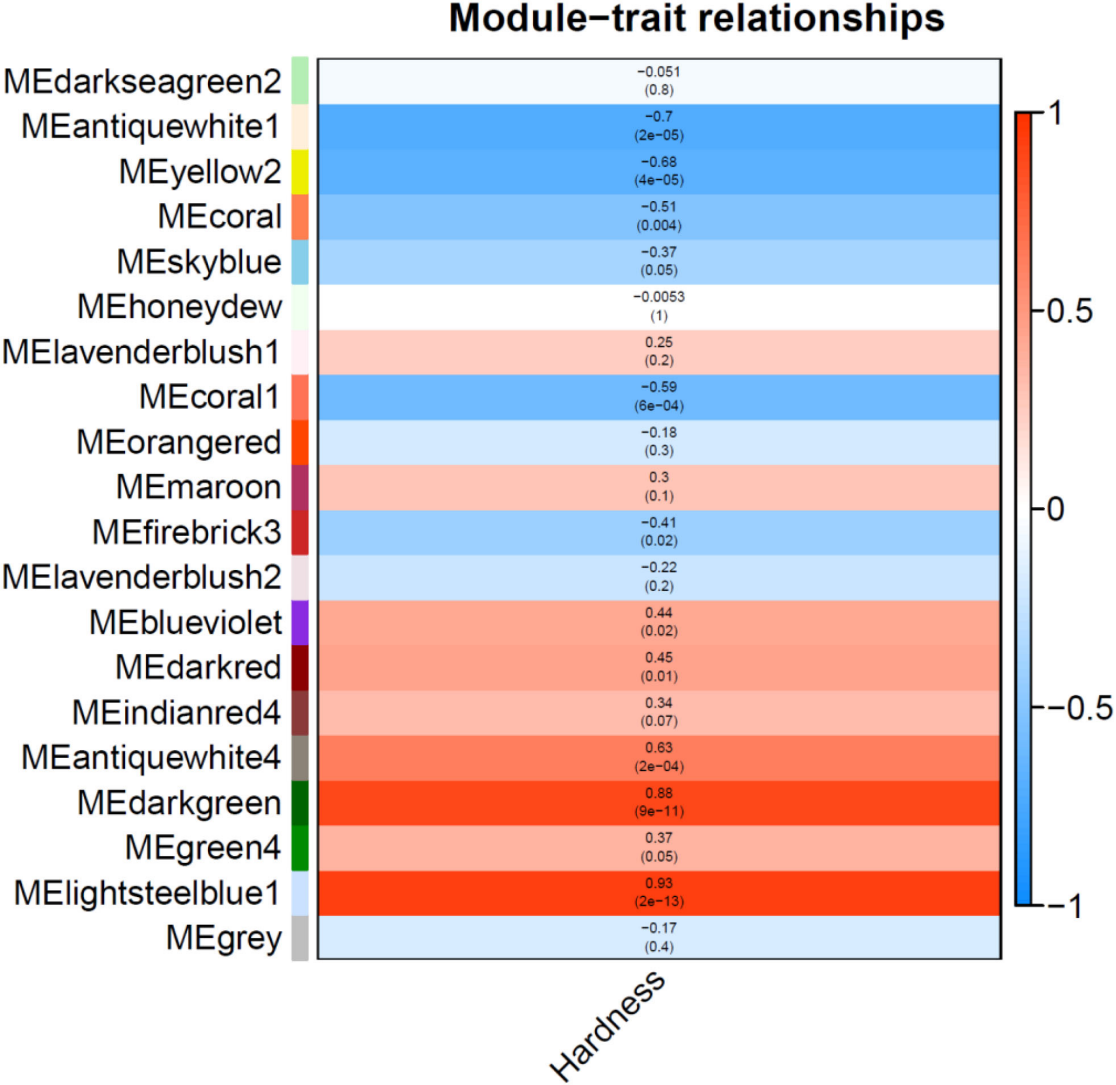
Module-trait correlation heatmap for strawberry hardness. The heatmap presents Pearson correlation coefficients (color scale) between gene co-expression modules (y-axis) and the hardness trait (x-axis). Each cell shows the correlation value with corresponding p-value in parentheses. Module names are indicated along the y-axis, with color intensity reflecting correlation strength (blue: -1, white: 0, red: +1).

**Figure 7** illustrates the correlation between gene co-expression modules and the phenotypic trait Hardness in strawberry fruits. The heatmap reveals significant associations (p < 0.05) across multiple modules, with the most striking being the MElightsteelblue1 module (r = 0.93, p = 2e-13) followed by MEdarkgreen (r = 0.88, p = 9e-11), showing a strong positive correlation with hardness, suggesting its genes may contribute to maintaining fruit firmness (**Supplementary Table S1**). Conversely, the MEantiquewhite1 (r = -0.7, p = 2e-05) and MEyellow2 (r = -0.68, p = 4e-05) modules exhibit strong negative correlations, implicating their roles in ripening-associated softening. Notably, the MEblueviolet module (r = 0.44, p = 0.02) and MEantiquewhite4 module (r = 0.63, p = 2e-04) also show moderate positive correlations, potentially linked to cell wall integrity or delayed ripening (**Figure 7**). These findings align with the phenotypic trends observed in **Figure 2**, where hardness declines sharply during the transfer color (T) to red (R) transition. The results highlight specific gene networks that could be targeted to modulate texture in strawberry breeding programs, with MElightsteelblue1 and MEdarkgreen as candidates for enhancing firmness, while MEantiquewhite1 and MEyellow2 may drive softening.

### 3.6 HCA analysis of hardness-related candidate genes in strawberry varieties

**Figure 8:**
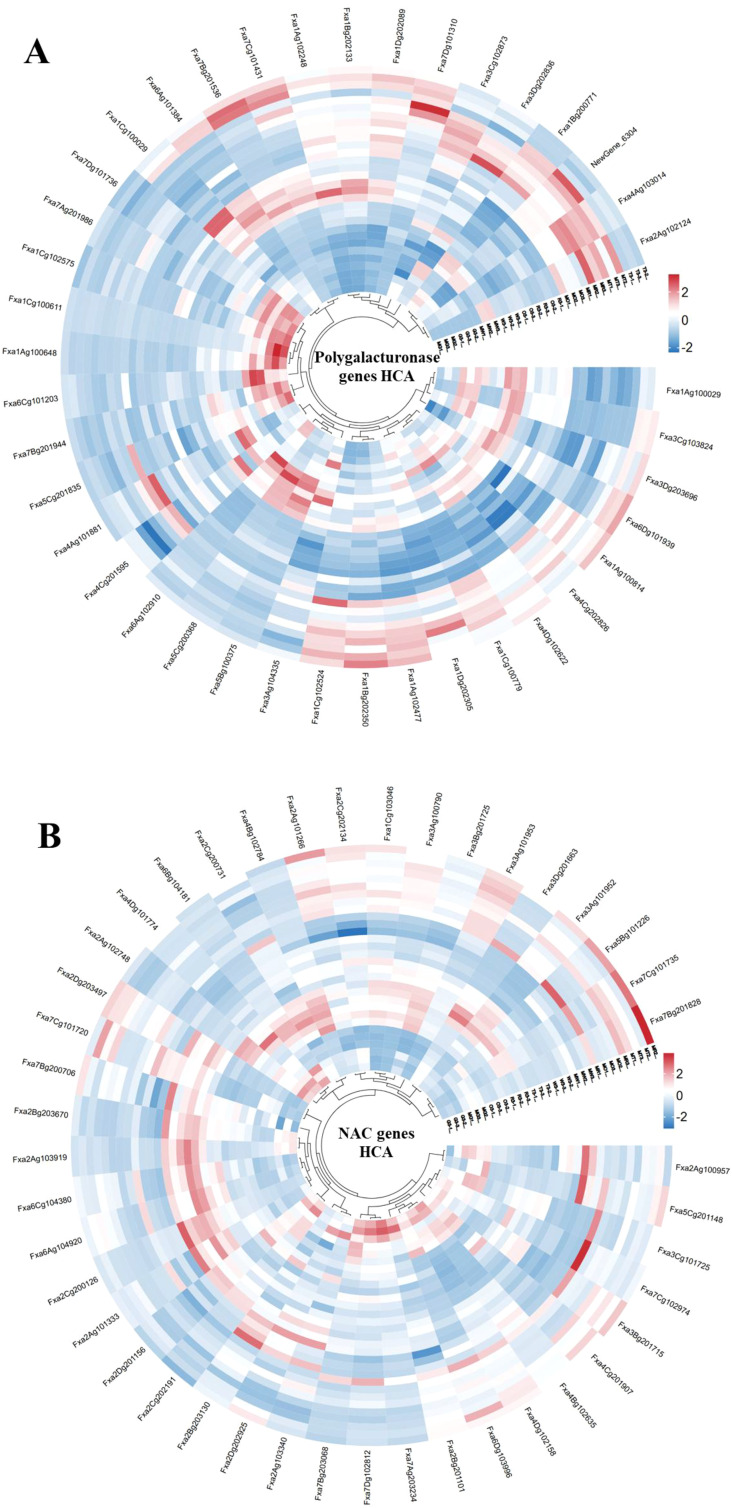
Hierarchical clustering analysis of stage-specific gene expression patterns in two strawberry varieties. Heatmaps depict normalized FPKM values (log2-transformed, fold change >2) for **(A)** polygalacturonase genes and **(B)** NAC transcription factors across five developmental stages (G: green; W: white; T: transfer color; R: red; O: overripe) in ‘Monterey’ (M) and ‘Three Princess’ (3). Rows represent genes; columns represent biological replicates per stage. Expression levels are z-score normalized (red: high expression; blue: low expression).

Hierarchical clustering analysis (HCA) of polygalacturonase genes revealed distinct expression patterns associated with fruit hardness across developmental stages in both strawberry varieties (**Figure 8A**). Among the forty polygalacturonase genes showing more than two-fold expression, four genes such as *Fxa1Ag102477*, *Fxa1Bg202350*, *Fxa1Cg102524*, and *Fxa7Dg101310*, were notably upregulated during the transfer color (T) stage in both ‘Three Princess’ and ‘Monterey’. These genes may encode enzymes involved in pectin degradation, a critical process for cell wall loosening during fruit softening. Their coordinated expression at the T stage aligns with the observed phenotypic softening (**Figure 2A**), suggesting their functional role in modulating textural changes during ripening.

Similarly, HCA of NAC transcription factors identified forty-three genes with differential expression patterns between the two varieties (**Figure 8B**). Notably, ‘Three Princess’ exhibited unique upregulation of *Fxa2Ag103919* and *Fxa6Cg104380* at the T and red (R) stages, which may contribute to its accelerated softening phenotype compared to ‘Monterey’. A striking varietal difference was observed for *Fxa2Dg203497*, which showed sustained high expression in ‘Monterey’ during the T, R, and overripe (O) stages but remained low in ‘Three Princess’. This differential expression pattern correlates with ‘Monterey’s’ firmer texture at later stages (**Figure 2A**), suggesting that the sustained expression of *Fxa2Dg203497* is associated with delayed softening in ‘Monterey’ (**Figure 8B**). These findings highlight the complex interplay between polygalacturonases and NAC TFs in determining strawberry fruit texture. The varietal differences in gene expression provide molecular insights into the contrasting hardness of ‘Three Princess’ and ‘Monterey’, offering potential targets for breeding programs aimed at optimizing fruit firmness.

**Figure 9:**
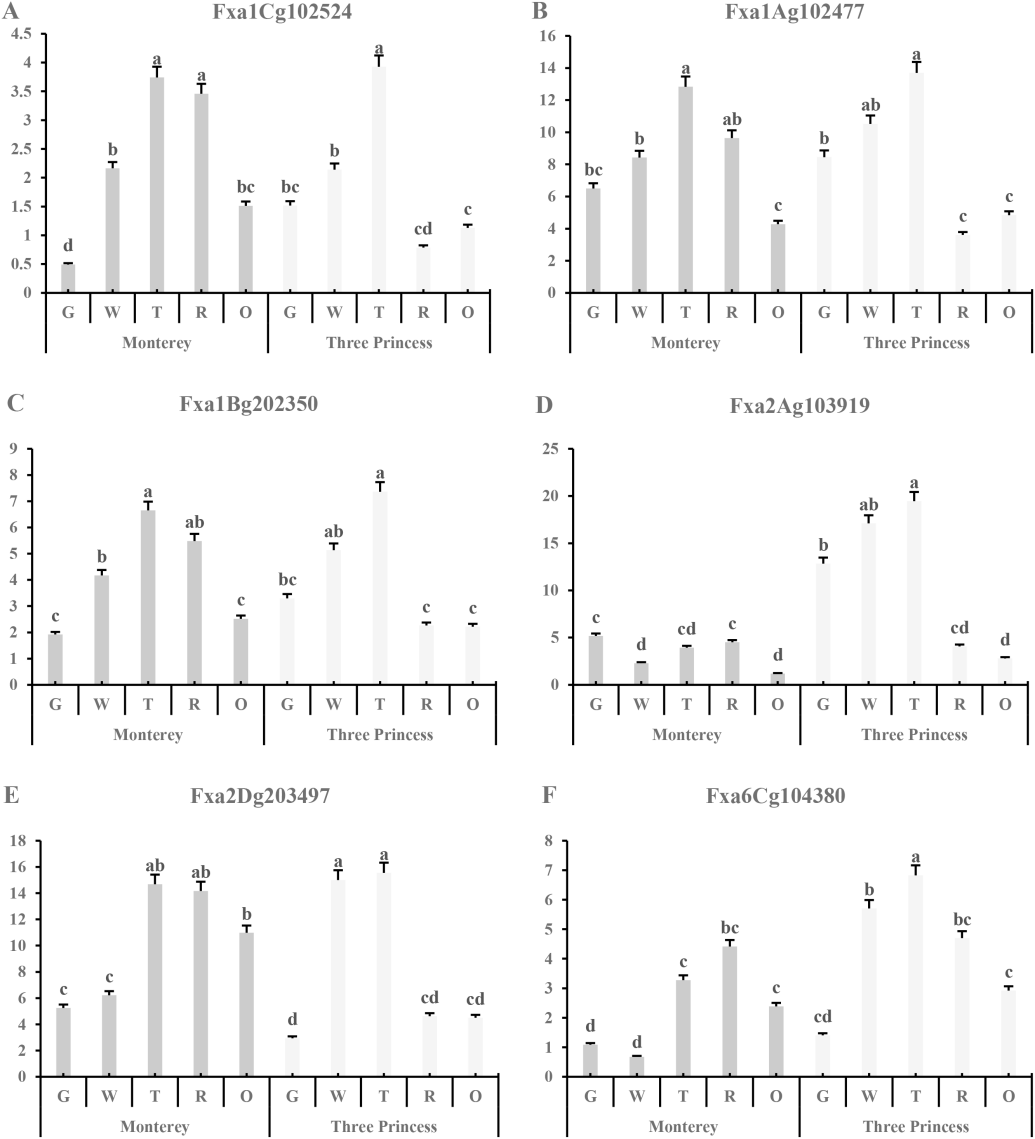
Expression patterns of candidate polygalacturonase and NAC transcription factor genes in ‘Monterey’ and ‘Three Princess’ strawberries across five developmental stages. **(A–C)** Expression profiles of polygalacturonase genes (*Fxa1Ag102477*, *Fxa1Bg202350*, *Fxa1Cg102524*). **(D–F)** Expression profiles of NAC transcription factor genes (*Fxa2Ag103919*, *Fxa6Cg104380*, *Fxa2Dg203497*). Gene expression levels are shown as mean FPKM values ± SE (n=3 biological replicates). Developmental stages: G, green; W, white; T, Transfer color; R, red; O, overripe.

Our analysis of key candidate genes revealed distinct expression dynamics that align with the contrasting softening phenotypes of the two varieties (**Figures 9A-F**). The polygalacturonase genes *Fxa1Ag102477* and *Fxa1Bg202350* showed a pronounced upregulation at the critical transfer color (T) stage in ‘Three Princess’, consistent with its rapid softening phenotype (**Figures 9B, C**). Conversely, the NAC transcription factor *Fxa2Dg203497* exhibited a striking varietal difference, with sustained high expression throughout ripening in ‘Monterey’ but consistently low expression in ‘Three Princess’ (**Figure 9E**). This pattern suggests a potential role for this NAC TF on the softening process, thereby contributing to ‘Monterey’s’ superior firmness retention. Another NAC gene, *Fxa2Ag103919*, was uniquely highly expressed in ‘Three Princess’ during early development, potentially priming its accelerated ripening program (**Figure 9D**). These expression patterns provide a molecular rationale for the observed textural differences, highlighting that the balance between cell wall degradation enzymes and their transcriptional regulators is a key determinant of final fruit firmness.

## 4 Discussion

The findings of this study provide a comprehensive understanding of the molecular mechanisms underlying the contrasting fruit quality traits in two strawberry cultivars, ‘Monterey’ and ‘Three Princess’. By integrating transcriptomic and phenotypic analyses, we uncovered stage-specific gene expression patterns that correlate with differences in fruit hardness and sweetness. These results align with and extend previous research on strawberry ripening (Aaby et al., 2012; Martín-Pizarro et al., 2021, Martín-Pizarro et al., 2025; Sánchez-Gómez et al., 2022; Lin et al., 2023; Yang et al., 2024), offering new insights into the genetic regulation of fruit quality. Our RNA-seq analysis revealed that the accelerated softening of ‘Three Princess’ was associated with early activation of polygalacturonases and starch/sucrose metabolism genes, consistent with previous studies highlighting the role of cell wall degradation enzymes in fruit texture (Xu et al., 2022; Zhao et al., 2024). The upregulation of polygalacturonases (e.g., *Fxa1Ag102477*, *Fxa1Bg202350*) during the transfer color stage in ‘Three Princess’ aligns with its rapid softening phenotype, mirroring findings in other soft-fruited cultivars (Hong et al., 2024; Jiménez et al., 2025). In contrast, ‘Monterey’ exhibited delayed expression of these genes, which correlates with its firmer texture. This observation supports the hypothesis that temporal regulation of cell wall-modifying enzymes is a key determinant of varietal differences in fruit hardness (Posé et al., 2011; Moya-León et al., 2019; Cai et al., 2021; Yuan et al., 2025).

The WGCNA analysis further identified co-expression modules strongly correlated with hardness, such as *MElightsteelblue1* (positively correlated) and *MEantiquewhite1* (negatively correlated) (**Supplementary Table S1**). These modules contained genes involved in cell wall remodeling and sugar metabolism, reinforcing the link between transcriptional networks and phenotypic traits. Similar co-expression patterns have been reported in tomato and apple, suggesting conserved mechanisms of fruit texture regulation across species (Janssen et al., 2008; Zenoni et al., 2023). Our results thus provide a robust framework for understanding how coordinated gene expression underpins textural changes during strawberry ripening.

A key finding was the varietal expression pattern of the NAC transcription factor *Fxa2Dg203497*, which showed sustained high expression in ‘Monterey’ but remained low in ‘Three Princess’. NAC TFs are known regulators of fruit ripening, with studies in tomato (*SlNOR*) and strawberry (*FaRIF*) demonstrating their role in modulating softening (Martín-Pizarro et al., 2021, Martín-Pizarro et al., 2025; Liu et al., 2024; Li et al., 2025). The sustained expression of *Fxa2Dg203497* in ‘Monterey’ suggests it may inhibit cell wall degradation, delay softening and extending shelf life. This aligns with previous work showing that overexpression of NAC TFs can enhance fruit firmness by repressing polygalacturonase activity (Liu et al., 2022). Our findings thus highlight *Fxa2Dg203497* as a promising candidate for breeding programs aimed at improving strawberry texture.

The higher SSC in ‘Three Princess’ was linked to earlier activation of starch/sucrose metabolism pathways, as evidenced by KEGG enrichment analysis (**Figures 2**, **5**). This cultivar exhibited stronger upregulation of genes involved in galactose metabolism and glycolysis, potentially facilitating faster sugar accumulation. Similar metabolic shifts have been observed in sweeter strawberry varieties, where early sucrose hydrolysis contributes to elevated SSC (Lee et al., 2018; Luo et al., 2020; Fan et al., 2021). The enrichment of fatty acid degradation pathways in ‘Three Princess’ further suggests a metabolic trade-off between energy mobilization and sugar storage, a phenomenon previously documented in ripening fruits (Liu et al., 2021; Zhu et al., 2022; Zhang et al., 2023). Interestingly, the hardness-to-SSC ratio revealed that ‘Three Princess’ maintained better textural stability despite its higher sugar content, a trait desirable for sensory quality. This contrasts with studies in other fruits, where increased sweetness often correlates with reduced firmness (Harker et al., 2006; Ketel et al., 2022). This finding challenges the prevailing model of a strict trade-off between sweetness and firmness in fleshy fruits. The concurrent maintenance of elevated soluble solids and textural integrity in ‘Three Princess’ suggests partial decoupling of the genetic programs governing sugar metabolism and cell wall remodeling. This cultivar-specific coordination of quality traits provides a valuable template for breeding strategies aimed at concurrently enhancing sweetness and postharvest durability.

The stage-resolved transcriptomic analysis uncovered distinct regulatory programs operating during early (green) and late (overripe) developmental stages. The green stage showed enrichment in flavonoid biosynthesis and pentose/glucuronate interconversions, indicative of active secondary metabolite production and cell wall assembly. These findings corroborate earlier work on strawberry fruit development, where early stages are characterized by cell division and expansion (Hussain et al., 2021; Sánchez-Gómez et al., 2022). The overripe stage, meanwhile, exhibited unique DEGs linked to senescence and stress responses, consistent with its role in postharvest deterioration (Belay and Caleb, 2022; Olennikov et al., 2022; Sirangelo et al., 2022; Quarshi et al., 2023; Priyadarshi et al., 2024). Our PCA and correlation analyses highlighted fundamental transcriptomic divergence between the two cultivars (**Figure 3**), with ‘Three Princess’ showing greater dynamic shifts during the transfer color to red transition. This transcriptional plasticity may be consistent with its rapid phenotypic progression, as reported in other fast-ripening berry fruits (Zapien-Macias et al., 2025). The conserved DEGs across all stages (2,018 genes) represent a core ripening regulon, potentially including master regulators of fruit maturation.

The differential expression of polygalacturonases and NAC TFs between ‘Monterey’ and ‘Three Princess’ provides actionable targets for marker-assisted breeding. For instance, allelic variants of *Fxa2Dg203497* associated with sustained expression could be selected for enhanced firmness, while modulating starch/sucrose metabolism genes may optimize sugar content. Future work should validate these candidates through functional studies, such as CRISPR-Cas9 or overexpression assays, as previously demonstrated (Ito et al., 2015; Rao and Wang, 2021; López-Casado et al., 2023). Integrating metabolomic and proteomic data could further elucidate post-transcriptional regulation of fruit quality. Additionally, expanding this approach to a broader range of cultivars would help distinguish conserved mechanisms from cultivar-specific adaptations. Such efforts would advance precision breeding strategies, enabling the development of strawberries with tailored texture and flavor profiles.

## 5 Conclusions

This study provides comprehensive insights into the molecular mechanisms underlying strawberry fruit quality by integrating phenotypic and transcriptomic analyses of two varieties with distinct hardness and sweetness profiles. Key findings show that ‘Three Princess’ undergoes accelerated softening due to early activation of polygalacturonases (e.g., *Fxa1Ag102477*) and starch/sucrose metabolism pathways, while ‘Monterey’ maintains firmer texture through sustained expression of NAC transcription factors such as *Fxa2Dg203497* and delayed cell wall degradation. Furthermore, WGCNA identified co-expression modules strongly correlated with hardness, pinpointing novel candidate genes associated with texture regulation. These results significantly advance our understanding of strawberry fruit development by demonstrating how stage-specific transcriptional reprogramming governs critical quality traits. The identification of key TFs and enzymatic genes provides a molecular framework for explaining varietal differences in ripening dynamics and postharvest performance.

Future research should focus on functional validation of candidate genes through transgenic approaches or genome editing. Moreover, expanding these findings to a broader range of strawberry genotypes could uncover conserved regulatory networks and cultivar-specific adaptations. Integrating metabolomic and proteomic data would further elucidate the post-transcriptional mechanisms influencing fruit quality. Ultimately, this work establishes a foundation for precision breeding strategies aimed at developing improved strawberry varieties with optimized texture, sweetness, and shelf life.

## Data Availability

The datasets presented in this study can be found in online repositories. The names of the repository/repositories and accession number(s) can be found below: https://db.cngb.org/data_resources/project/CNP0007866/, CNP0007866.
