# Supplementary material for: Integrative transcriptomics and phenotyping uncover genetic networks controlling fruit quality in two strawberries (Fragaria × ananassa)
Source: Front Plant Sci. 2026 Jan 19;16:1700348. doi: 10.3389/fpls.2025.1700348 (PMC12862081; doi:10.3389/fpls.2025.1700348)
Supplement: Supplementary file 1 [file Image1.pdf]

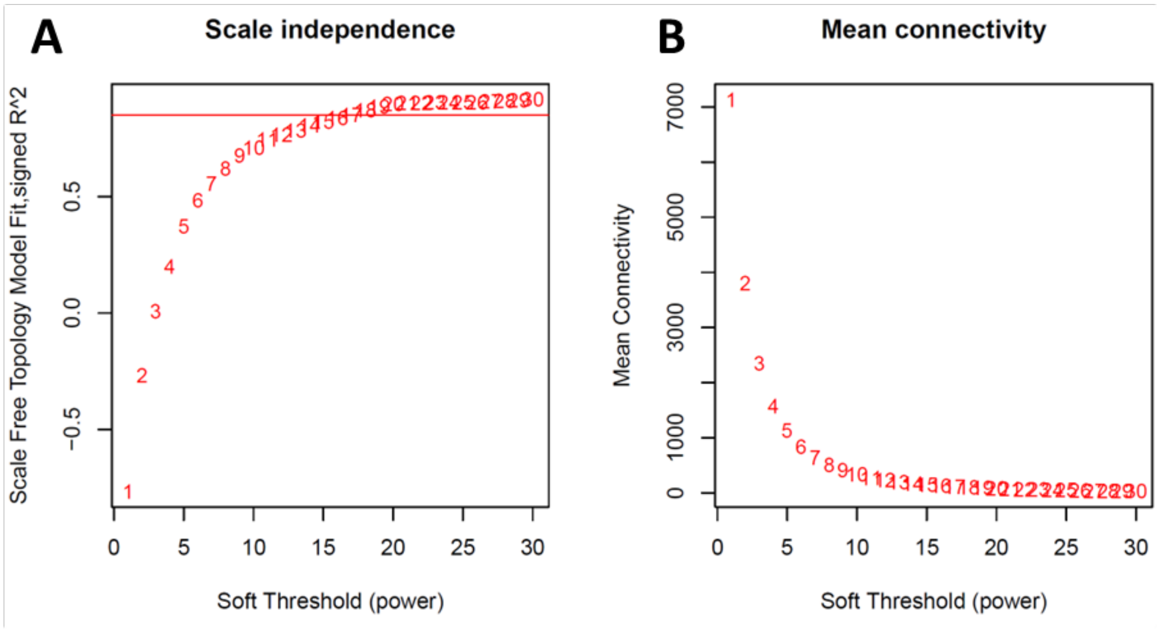

Supplementary Figure S1. Analysis of network topology for various soft-thresholding powers. (A) Scale-free topology model fit (signed R^2) as a function of the soft-thresholding power. The chosen power (β=17) is where the scale-free topology fit curve flattens out at a high value ($R^2 > 0.85$). (B) Mean connectivity as a function of the soft-thresholding power.

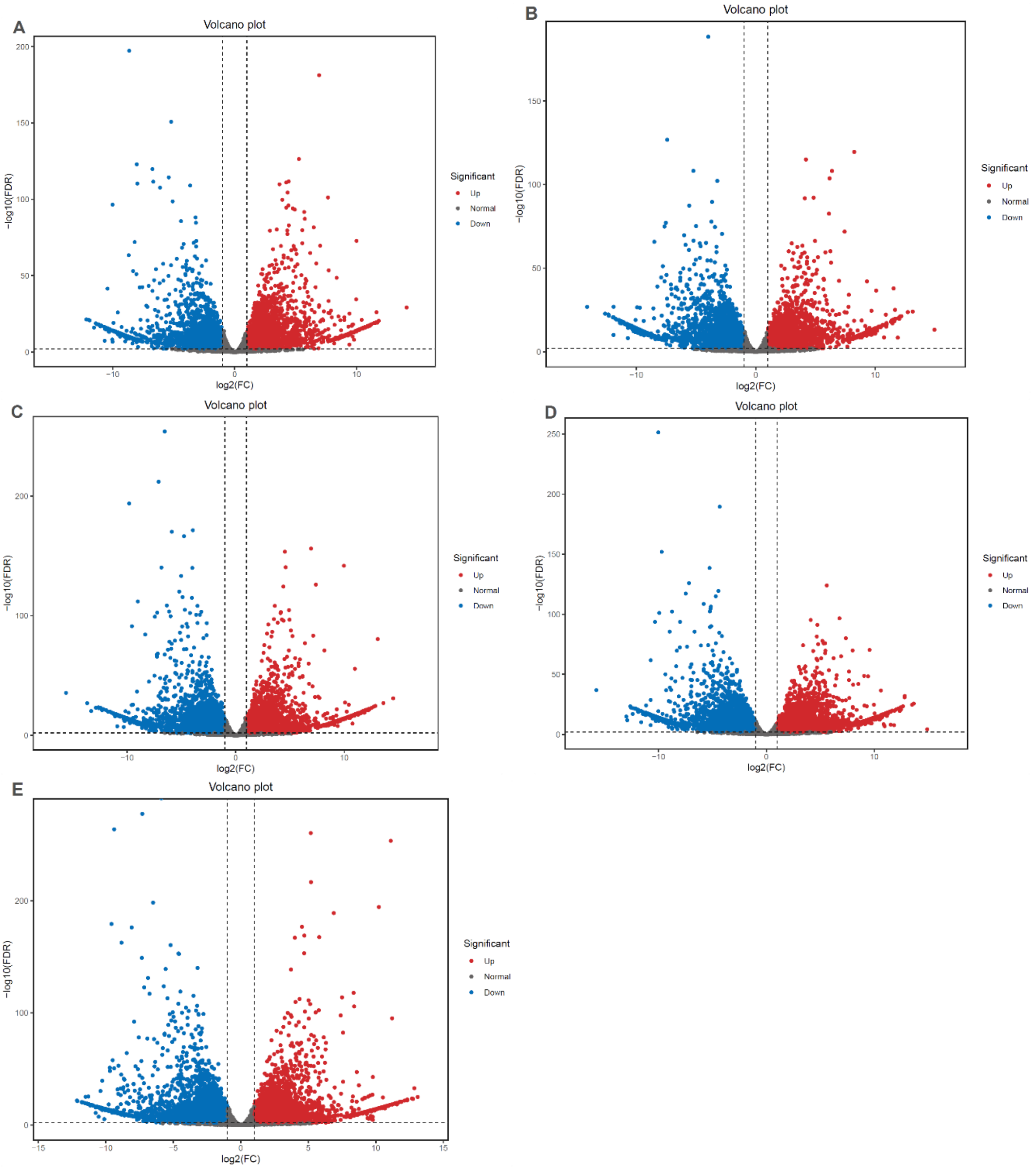

Supplementary Figure S2. Volcano plots visualizing differential expression between 'Three Princess' and 'Monterey'. Each plot displays the log2 fold-change (x-axis) against the -log10(FDR) (y-axis) for genes in paired comparisons at the same developmental stage. Significantly upregulated (red) and downregulated (blue) genes in 'Three Princess' are identified using a threshold of FDR < 0.05 and $|\log2FC| \geq 1$. The stages compared are: (A) Green (3G vs MG), (B) White (3W vs MW), (C) Transfer Color (3T vs MT), (D) Red (3R vs MR), and (E) Overripe (3O vs MO).
